# Prevalence and Associated Echocardiographic Findings of Pulmonary Hypertension in Chronic Kidney Disease: A Cross-Sectional Study

**DOI:** 10.7759/cureus.108609

**Published:** 2026-05-10

**Authors:** K. Tamilselvan, Aashish Arumugam, Magesh Vadivelu, K. Senthilkumar

**Affiliations:** 1 Department of Cardiology, Meenakshi Medical College Hospital and Research Institute, Meenakshi Academy of Higher Education and Research, Kanchipuram, IND; 2 Department of Nephrology, Meenakshi Medical College Hospital and Research Institute, Meenakshi Academy of Higher Education and Research, Kanchipuram, IND

**Keywords:** chronic kidney disease, echocardiography, pulmonary hypertension, right atrial pressure, tapse, tricuspid regurgitation velocity

## Abstract

Background: Pulmonary hypertension (PH) is an increasingly recognized cardiovascular complication in chronic kidney disease (CKD), yet the echocardiographic features associated with its presence remain insufficiently characterized in routine nephrology practice. This study determined the prevalence of echocardiographically defined PH in CKD and examined echocardiographic variables associated with its presence.

Methodology: This cross-sectional study included 120 adults with stages 3-5 CKD, including both conservatively managed patients and those receiving maintenance hemodialysis, evaluated between November 2024 and December 2025. PH was defined echocardiographically as an estimated pulmonary artery systolic pressure (PASP) >35 mmHg. Clinical, renal, and echocardiographic variables were compared between patients with and without PH. Because the estimated PASP was derived from tricuspid regurgitation velocity (TRV) and estimated right atrial pressure (RAP), these variables were considered definitional components of PH classification rather than independent associated findings. Exploratory analyses focused on non-definitional clinical and echocardiographic variables.

Results: PH was present in 68 (56.7%) patients, while 52 (43.3%) had no PH. Mild PH was observed in 21 (17.5%), moderate PH in 25 (20.8%), and severe PH in 22 (18.3%). Patients with PH more frequently had stage 5 CKD (40, 58.8%, vs. 14, 26.9%), dialysis dependence (38, 55.9%, vs. 16, 30.8%), volume overload (41, 60.3%, vs. 16, 30.8%), and diastolic dysfunction (53, 77.9%, vs. 22, 42.3%). Among non-definitional echocardiographic findings, patients with PH had lower tricuspid annular plane systolic excursion, larger right atrial diameter, and more frequent diastolic dysfunction. TRV and estimated RAP were not interpreted as independent associated variables because they contributed directly to PASP estimation.

Conclusions: PH was present in 68 of 120 patients with CKD, corresponding to a prevalence of 56.7%. Because PH was classified using estimated PASP derived from TRV and estimated RAP, these variables were not interpreted as independent echocardiographic correlates. Non-definitional echocardiographic findings associated with PH included lower tricuspid annular plane systolic excursion, larger right atrial size, and more frequent diastolic dysfunction. These findings support careful multiparameter echocardiographic assessment in CKD but should be interpreted as associative rather than predictive evidence.

## Introduction

Chronic kidney disease (CKD) is increasingly recognized as a systemic disorder characterized not only by progressive renal dysfunction but also by substantial cardiovascular morbidity and mortality [1]. Cardiovascular disease accounts for nearly half of all deaths in patients with advanced CKD, reflecting complex interactions between renal impairment, hemodynamic alterations, neurohormonal activation, and structural cardiac remodeling [2]. Among the diverse cardiovascular complications associated with CKD, pulmonary hypertension (PH) has emerged as an important yet frequently underrecognized entity that contributes to worsening functional status, right ventricular (RV) dysfunction, and adverse clinical outcomes. Although PH has traditionally been studied in pulmonary vascular disease and left heart disorders, accumulating evidence indicates that CKD itself may constitute a distinct pathophysiological milieu predisposing to pulmonary vascular and right heart abnormalities [3].

The reported prevalence of PH in CKD varies widely depending on the stage of renal disease, patient selection, and diagnostic methodology. Population-based cohort analyses have demonstrated that PH occurs in approximately 21% of patients with nondialysis CKD and increases progressively with worsening renal function [4]. Similarly, large observational analyses have reported prevalence estimates ranging from 26% to nearly 50% in moderate-to-advanced CKD populations [5]. Meta-analytic synthesis of observational data further suggests that the pooled prevalence of PH across the CKD spectrum approaches 38%, with even higher rates observed among patients receiving maintenance hemodialysis [6]. These findings collectively indicate that pulmonary vascular involvement represents a frequent cardiorenal complication rather than a rare incidental finding.

Echocardiography has become the principal noninvasive modality for screening and initial evaluation of PH in CKD populations. Doppler-derived estimates of pulmonary artery systolic pressure (PASP) obtained from tricuspid regurgitation velocity (TRV) provide a practical and widely used method for identifying elevated pulmonary pressures in clinical practice [7]. Additional echocardiographic parameters, including right atrial (RA) size, tricuspid annular plane systolic excursion (TAPSE), and indices of left ventricular diastolic dysfunction, provide complementary information regarding RV function and the underlying hemodynamic phenotype of PH [8]. Systematic evaluations have demonstrated that combinations of echocardiographic parameters such as TRV, right atrial pressure (RAP), and RV functional indices substantially improve the ability to identify clinically significant PH compared with single measurements alone [9].

Beyond its diagnostic implications, PH in CKD also carries important prognostic significance. Prospective cohort studies have shown that elevated PASP independently predicts cardiovascular events and mortality in predialysis CKD populations [4,10]. In dialysis populations, the presence of PH has similarly been associated with increased hospitalization and reduced survival [11]. These observations suggest that PH represents not merely a hemodynamic abnormality but a clinically meaningful marker of cardiovascular vulnerability within the CKD population. Against this background, the primary objective of the study was to determine the prevalence of echocardiographically defined PH in adults with stage 3 to stage 5 CKD. The secondary objectives were to compare demographic, renal, clinical, and non-definitional echocardiographic findings between patients with and without PH and to explore whether CKD severity modified selected associations.

## Materials and methods

This study was conducted in the Department of Nephrology at Meenakshi Medical College Hospital and Research Institute (MMCHRI) from November 2024 to December 2025. The study was initiated after obtaining ethical approval from the Institutional Ethics Committee (IEC) (MMCHRI/IEC/PG/11/10/2024). The study was designed within a cross-sectional framework to determine the burden of echocardiographically defined PH among patients with CKD and to identify the clinical renal and cardiac functional variables associated with its presence.

Study population

A total of 120 patients with established CKD were included in the final analysis. Adult patients aged 18 years and above with CKD stage 3, stage 4, or stage 5 who underwent transthoracic echocardiographic evaluation during the study period were considered eligible. Both patients managed conservatively, and those receiving maintenance hemodialysis were included so that the cohort would reflect the full clinical spectrum of moderate-to-advanced renal dysfunction encountered in routine nephrology practice. Patients with incomplete clinical laboratory or echocardiographic data relevant to the study variables were excluded. In patients who had undergone more than one echocardiographic examination during the study window, the index examination temporally closest to the clinical evaluation was selected for analysis.

Clinical and renal assessment

Demographic and clinical data were obtained from case records and bedside assessment at the time of enrollment. Age was recorded as a continuous variable and was additionally categorized into clinically relevant strata for descriptive presentation. CKD was staged according to standard nephrology staging criteria and classified as stage 3, stage 4, or stage 5. Dialysis status was recorded as maintenance hemodialysis versus no dialysis. Clinical evidence of volume overload was documented on routine physician assessment and included features such as pedal edema, elevated jugular venous pressure, pulmonary congestion, or related signs suggestive of fluid excess. Hemoglobin concentration was recorded from the laboratory value closest to the echocardiographic examination to preserve temporal comparability between hematologic and hemodynamic assessment.

Echocardiographic assessment

All patients underwent transthoracic echocardiography using standard adult imaging windows and routine departmental measurement protocols with a Philips X5-1c transducer (Philips Healthcare, Andover, MA). Examinations were performed and interpreted by two cardiologists, each with 12 years of experience in echocardiography, using blinded assessment techniques. The principal study outcome was PH estimated noninvasively from PASP. PASP was derived from tricuspid regurgitation velocity using the modified Bernoulli equation with the addition of estimated RAP. Therefore, tricuspid regurgitation velocity and estimated RAP were treated as components of PASP estimation and PH classification, and were not interpreted as independent echocardiographic features, correlates, or predictors of PH. PH was defined as an estimated PASP >35 mmHg.

For descriptive analysis, PH severity was categorized as mild when PASP was 36 to 45 mmHg, moderate when it was 46 to 60 mmHg, and severe when it exceeded 60 mmHg. Right-sided cardiac structure and function were evaluated using RA diameter and tricuspid annular plane systolic excursion. Tricuspid regurgitation velocity and estimated RAP were used only for PASP estimation and PH classification. RA size was categorized descriptively as normal when less than 40 mm, mild enlargement when 40 to 49 mm, and moderate-to-severe enlargement when 50 mm or greater. Tricuspid annular plane systolic excursion was used as an index of right ventricular longitudinal systolic function. Left ventricular diastolic function was assessed according to standard echocardiographic criteria and graded as grade 0, grade I, grade II, or grade III dysfunction for descriptive reporting. For regression analysis, diastolic dysfunction was additionally handled as a binary variable, present or absent, to enhance interpretability and preserve model stability.

**Figure 1:**
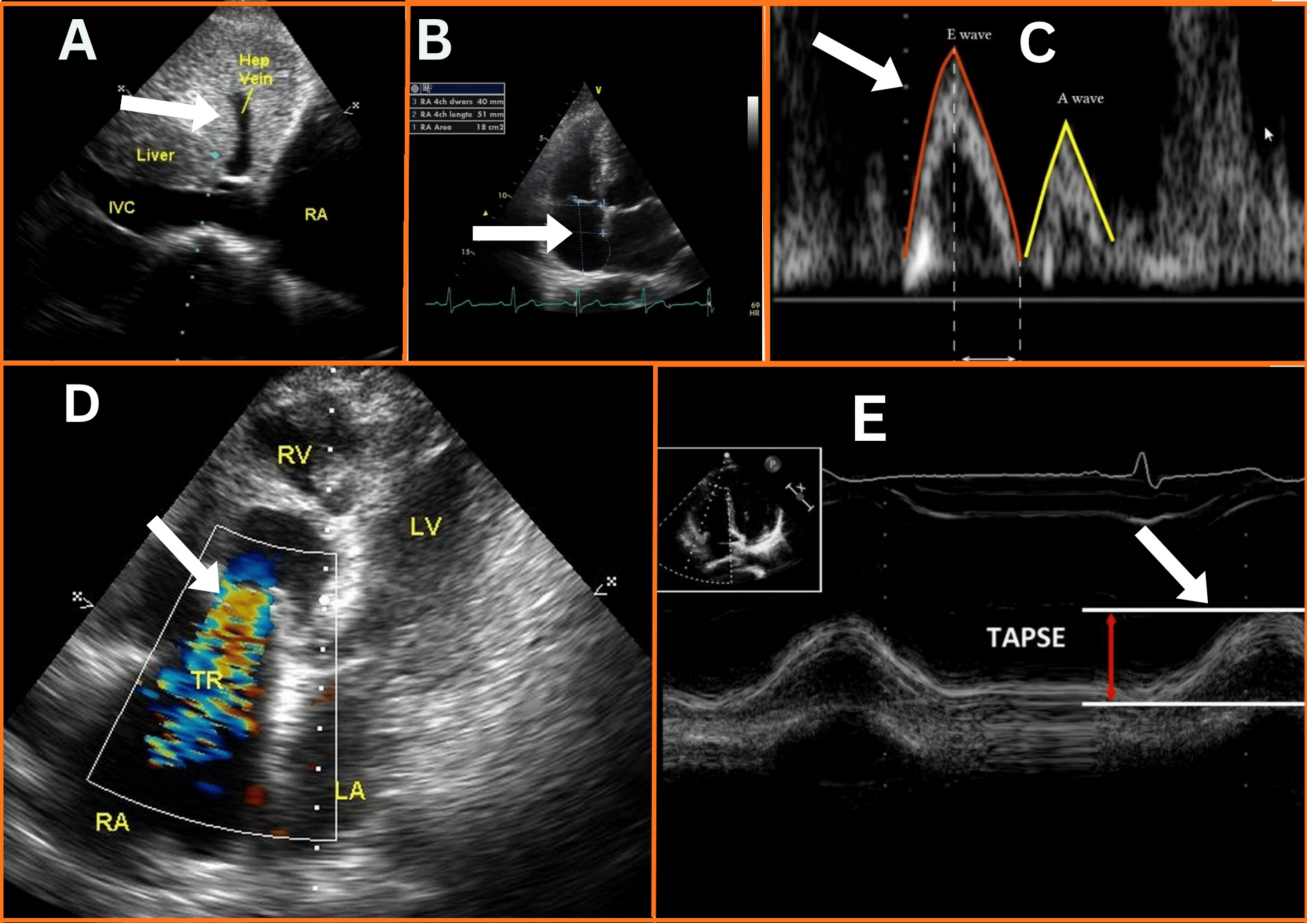
Representative echocardiographic measurements used in the assessment of pulmonary hypertension in chronic kidney disease. (A) Subcostal echocardiographic view showing the inferior vena cava and hepatic vein for assessment of right atrial pressure based on inferior vena cava diameter and inspiratory collapse. (B) Apical four-chamber view demonstrating right atrial enlargement. (C) Pulsed-wave Doppler tracing of transmitral inflow showing the E and A waves. The E wave represents early passive left ventricular filling, whereas the A wave represents atrial contraction during late diastolic filling. (D) Color Doppler image in the apical four-chamber view showing tricuspid regurgitation across the tricuspid valve.

For interpretive context, standard reference ranges were stated for all principal echocardiographic variables used in the analysis. A TRV value below 2.8 m/s was considered within the usual reference range, while higher values increased the echocardiographic probability of PH. Estimated RAP was interpreted according to standard inferior vena cava size and collapsibility criteria and generally categorized as 3, 8, or 15 mmHg. TAPSE ≥17 mm was considered within the normal range for right ventricular longitudinal systolic function. RA dimensions were interpreted against standard right-heart reference values. Because the present study used RA diameter, the measurement view and cardiac phase were specified to ensure reproducibility. PASP values up to approximately 35 mmHg were considered non-elevated for the operational purposes of this study (Figure 1).

Outcome measures

The primary objective of the study was to determine the prevalence of echocardiographically defined PH in patients with CKD. The primary outcome was the presence or absence of PH as defined by echocardiography. The secondary objectives were to describe the severity distribution of PH within the cohort, compare demographic, renal, clinical, and echocardiographic characteristics between patients with and without PH, examine echocardiographic variables associated with PH classification, explore whether CKD severity modified selected associations, and assess the apparent within-cohort discriminatory performance of sequential exploratory models incorporating echocardiographic parameters.

Statistical analysis

Data were analyzed using IBM SPSS Statistics for Windows, version 23.0 (IBM Corp., Armonk, NY). Categorical variables were summarized as frequencies and percentages, and continuous variables as mean ± standard deviation. Group comparisons between patients with and without PH were performed using the chi-square test or Fisher's exact test for categorical variables and the independent-samples t-test for continuous variables after assessment of approximate normality. A two-tailed *P*-value <0.05 was considered statistically significant.

Binary logistic regression was performed with PH status as the dependent variable. Univariate models were initially fitted for clinically relevant renal and echocardiographic variables. Continuous predictors were scaled in clinically meaningful units: RA diameter per 5 mm increase and tricuspid annular plane systolic excursion per 1 mm decrease. TRV and estimated RAP were not included as independent predictors because they were used to derive PASP and classify PH. The revised regression analysis was limited to univariate models after excluding tricuspid regurgitation velocity and estimated RAP as independent predictors because both variables contributed directly to PASP estimation and PH classification. Odds ratios were reported with 95% confidence intervals.

Interaction terms between CKD stage and selected echocardiographic variables were tested to explore effect modification. Patients with incomplete key clinical, laboratory, or echocardiographic data were excluded, and complete-case analysis was performed.

## Results

A total of 120 patients with CKD were evaluated. The cohort was predominantly middle-aged to elderly. Patients aged 40-59 years accounted for 51/120 (42.5%), and those aged 60-74 years accounted for 50/120 (41.7%), whereas only 9/120 (7.5%) were younger than 40 years and 10/120 (8.3%) were aged 75 years or older. Advanced renal dysfunction was common, with stage 5 CKD present in 54/120 (45.0%) patients, stage 4 CKD in 37/120 (30.8%), and stage 3 CKD in 29/120 (24.2%). Maintenance hemodialysis was being received by 54/120 (45.0%) patients, while 66/120 (55.0%) were not on dialysis (Table 1).

**Table 1:** Baseline demographic and renal characteristics of the study population (N = 120). Data are presented as *n* (%). This table is descriptive; no inferential statistical test was applied.

Variable	*n* (%)
Age group (years)	
<40	9 (7.5)
40- 59	51 (42.5)
60-74	50 (41.7)
≥75	10 (8.3)
CKD stage	-
Stage 3	29 (24.2)
Stage 4	37 (30.8)
Stage 5	54 (45.0)
Dialysis status	-
No	66 (55.0)
Yes, maintenance hemodialysis	54 (45.0)

PH, defined by an estimated PASP greater than 35 mmHg, was present in 68/120 (56.7%) patients. Among the entire cohort, 21/120 (17.5%) had mild PH, 25/120 (20.8%) had moderate PH, and 22/120 (18.3%) had severe PH, while 52/120 (43.3%) had no PH. RA enlargement was frequent, with normal RA size in 40/120 (33.3%), mild enlargement in 37/120 (30.8%), and moderate-to-severe enlargement in 43/120 (35.8%). Diastolic dysfunction was identified in 75/120 (62.5%) patients, comprising grade I dysfunction in 28/120 (23.3%), grade II in 23/120 (19.2%), and grade III in 24/120 (20.0%). Clinical signs of volume overload were observed in 57/120 (47.5%) patients (Table 2).

**Table 2:** Cardiovascular and echocardiographic characteristics of the study population (N = 120). Data are presented as* n *(%). PASP indicates PASP. This table is descriptive; no inferential statistical test was applied.

Variable	*n* (%)
Pulmonary hypertension by PASP	
Normal, ≤35 mmHg	52 (43.3)
Mild, 36-45 mmHg	21 (17.5)
Moderate, 46-60 mmHg	25 (20.8)
Severe, >60 mmHg	22 (18.3)
Right atrial size	
Normal, <40 mm	40 (33.3)
Mild enlargement, 40-49 mm	37 (30.8)
Moderate-to-severe enlargement, ≥50 mm	43 (35.8)
Grade of diastolic dysfunction	
Grade 0	45 (37.5)
Grade I	28 (23.3)
Grade II	23 (19.2)
Grade III	24 (20.0)
Clinical signs of volume overload	
No	63 (52.5)
Yes	57 (47.5)

When stratified by PH status, the mean age was similar between patients with and without PH (59.8 ± 12.8 vs. 57.6 ± 12.5 years, *P* = 0.34). In contrast, markers of more advanced kidney disease and greater hemodynamic burden were substantially more frequent among patients with PH. Stage 5 CKD was present in 40/68 (58.8%) patients with PH compared with 14/52 (26.9%) without PH (*P* < 0.001), and dialysis dependence was also more common in the PH group (38/68, 55.9%, vs. 16/52, 30.8%, *P* = 0.006). Patients with PH had lower hemoglobin concentrations and more frequent clinical volume overload Among non-definitional echocardiographic findings, PH was associated with lower TAPSE, larger RA diameter, and a markedly greater prevalence of diastolic dysfunction. TRV and estimated RAP were higher in the PH group as expected, because they contributed directly to PASP estimation and PH classification (Table 3).

**Table 3:** Comparison of patients with and without PH. Data are presented as mean ± standard deviation (SD) or *n* (%), as appropriate. Continuous variables were compared using the independent-samples t-test, and categorical variables were compared using the chi-square test or Fisher's exact test, as appropriate. A two-tailed *P*-value < 0.05 was considered statistically significant, and significant *P*-values are marked with *.\ CKD, chronic kidney disease; TAPSE, tricuspid annular plane systolic excursion; RA, right atrial

Characteristic	Total cohort (*N* = 120)	PH present (*n* = 68)	PH absent (*n* = 52)	Test statistic	*P*-value
Age, years, mean ± SD	58.8 ± 12.7	59.8 ± 12.8	57.6 ± 12.5	*t* = 0.94	0.34
CKD stage 5, *n* (%)	54 (45.0)	40 (58.8)	14 (26.9)	*χ*² = 12.12	<0.001*
On dialysis, *n* (%)	54 (45.0)	38 (55.9)	16 (30.8)	*χ*² = 7.51	0.006*
Hemoglobin, g/dL, mean ± SD	11.3 ± 1.7	10.5 ± 1.4	12.1 ± 1.5	*t* = 6.01	<0.001*
Volume overload, *n* (%)	57 (47.5)	41 (60.3)	16 (30.8)	*χ*² = 10.30	0.002*
TAPSE, mm, mean ± SD	17.9 ± 3.4	16.1 ± 2.4	20.2 ± 3.1	*t* = 8.17	<0.001*
RA diameter, mm, mean ± SD	45.3 ± 7.6	49.2 ± 6.4	40.2 ± 5.8	*t* = 7.95	<0.001*
Diastolic dysfunction present, *n* (%)	75 (62.5)	53 (77.9)	22 (42.3)	*χ*² = 15.96	<0.001*

On univariate logistic regression, several variables were significantly associated with PH. After excluding parameters used for PASP estimation, lower TAPSE, larger RA diameter, the presence of diastolic dysfunction, dialysis dependence, and stage 5 CKD were associated with PH on unadjusted analysis (Table 4).

**Table 4:** Univariate logistic regression for variables associated with PH. Odds ratios with 95% confidence intervals (CIs) were derived from binary univariate logistic regression models, with PH as the dependent variable. The Wald chi-square statistic was applied for each predictor. A two-tailed *P*-value < 0.05 was considered statistically significant, and significant *P*-values are marked with *. TAPSE, tricuspid annular plane systolic excursion; RA, right atrium; CKD, chronic kidney disease; PH, pulmonary hypertension

Predictor	Odds ratio	95% CI	Wald χ²	*P*-value
TAPSE, per 1 mm decrease	1.84	1.42-2.43	19.80	<0.001*
RA diameter, per 5 mm increase	2.88	1.98-4.31	28.42	<0.001*
Diastolic dysfunction, present vs. absent	4.78	2.17-10.87	14.49	<0.001*
Dialysis, yes vs. no	2.85	1.34-6.08	7.37	0.006*
CKD stage 5, yes vs. no	3.88	1.83-8.31	12.34	<0.001*

Interaction analysis suggested that CKD severity modified the association of TAPSE and diastolic dysfunction with PH, as reflected by statistically significant interaction terms. No significant interaction was observed between CKD stage and RA diameter (Table 5).

**Table 5:** Effect modification by CKD severity. The Wald chi-square statistic was applied for each interaction term. A two-tailed *P*-value < 0.05 was considered statistically significant, and significant *P*-values are marked with *. TAPSE, tricuspid annular plane systolic excursion; RA, right atrium; CKD, chronic kidney disease

Interaction term	Wald χ²	*P*-value for interaction
CKD stage × TAPSE	4.18	0.041*
CKD stage × RA diameter	2.32	0.128
CKD stage × diastolic dysfunction	5.09	0.024*

## Discussion

This study shows that PH is not an incidental echocardiographic abnormality in CKD, but a frequent and clinically meaningful cardiorenal complication. More than half of the cohort had PH, and the burden was not confined to mild elevations alone, as a substantial proportion already had moderate or severe disease. The central finding, however, lies less in prevalence alone and more in the pattern of association. PH clustered with stage 5 CKD, dialysis dependence, lower hemoglobin, clinical volume overload, larger right atrial diameter, lower TAPSE, and more frequent diastolic dysfunction. TRV and estimated RAP were higher in patients with PH, as expected, because they contributed directly to PASP estimation and PH classification; therefore, they were not interpreted as independent echocardiographic findings. This suggests that the hemodynamic and structural consequences of advanced kidney disease may be more proximate determinants of PH than dialysis exposure itself.

The prevalence observed in this cohort is higher than that reported in several nondialysis CKD studies, such as Navaneethan and Dweik [4] and Bolignano et al. [10], where PH was present in approximately one-fifth to one-fourth of patients, who reported a prevalence of 26.6%. Yet the present estimate is broadly consistent with studies enriched for advanced disease and dialysis exposure. Zhang et al. [5] reported a prevalence of 47.38% across the CKD spectrum, Gaur et al. [12] found PH in 47%, Nagaraju et al. [13] reported 54% in maintenance hemodialysis patients, and Lin et al. [6] estimated a pooled prevalence of 38% overall, rising further in hemodialysis populations. The higher frequency in the present study is therefore plausible and likely reflects the composition of the sample, which was dominated by stages 4 and 5 CKD and included a large proportion of dialysis patients. This pattern reinforces a recurring theme in the literature: the burden of PH rises as renal dysfunction advances.

The association between PH and advanced CKD severity in this study is strongly aligned with prior work. Navaneethan and Dweik [4] demonstrated a graded rise in PH with worsening CKD stage, while Zhang et al. [5] showed a similar escalation across the renal spectrum. O'Leary et al. [14], using invasive hemodynamic data, also confirmed that PH becomes more common as CKD progresses. The present findings fit well within this framework. Declining kidney function likely creates a permissive substrate through chronic salt and water retention, anemia, endothelial dysfunction, vascular calcification, neurohormonal activation, and left ventricular remodeling. Di Lullo et al. [15] and Sise et al. [3] both emphasized that PH in CKD is rarely attributable to a single mechanism. Rather, it emerges from the cumulative interaction of volume stress, cardiac dysfunction, altered vascular biology, and, in some patients, dialysis-related hemodynamic factors.

The echocardiographic interpretation of the present study requires caution. TRV and estimated RAP were used to derive PASP and therefore formed part of the operational classification of PH. From an imaging standpoint, these parameters should not be presented as independent predictors or correlates of PH. The more clinically meaningful non-definitional findings were lower TAPSE, larger right atrial diameter, and more frequent diastolic dysfunction. These findings suggest right ventricular longitudinal functional impairment, right-sided chamber remodeling, and possible contribution of left-sided filling pressure or volume-related mechanisms in CKD-associated PH.

The strong association with diastolic dysfunction also deserves emphasis. Patients with PH had a markedly greater prevalence of diastolic dysfunction, and CKD stage modified this relationship. This is in keeping with the invasive observations of O'Leary et al. [14], who found that postcapillary PH predominated in CKD, and with Ramasubbu et al. [11], who linked pulmonary pressures in hemodialysis patients to elevated wedge pressure and left-sided dysfunction. Taken together, these data support the interpretation that much of PH in CKD may reflect elevated left-sided filling pressures and chronic congestion rather than isolated pulmonary vascular disease.

Some findings, however, diverge from parts of the literature. Age was not significantly associated with PH in this cohort, whereas older age emerged as a predictor in Bolignano et al. [10], Navaneethan and Dweik [4], and Hu et al. [16]. A plausible explanation is that, in a cohort already enriched with advanced CKD and hemodynamic stress, renal and cardiac variables may overshadow the relatively weaker independent effect of age. Likewise, dialysis was significant in univariate analysis; however, this association should be interpreted cautiously because dialysis status may reflect the broader hemodynamic burden of advanced CKD rather than dialysis exposure alone. This apparent disagreement with studies emphasizing dialysis as an independent correlate may actually indicate mediation rather than contradiction. Dialysis patients often have greater fluid shifts, more advanced myocardial remodeling, and higher right-heart burden; therefore, the observed association between dialysis and PH may reflect the broader hemodynamic burden of advanced CKD rather than dialysis exposure alone.

This study has several limitations. Its cross-sectional design precludes causal inference and limits conclusions to association rather than temporal prediction. The study was conducted at a single center with a modest sample size, which may limit generalizability and increase the risk of model overfitting. No formal external validation was performed, and the findings should therefore be interpreted as descriptive and internally associative. PH was defined echocardiographically rather than by right-heart catheterization, which may have introduced misclassification. In addition, tricuspid regurgitation velocity and estimated right atrial pressure contributed directly to the calculation of estimated PASP and were therefore not interpreted as independent associated variables. Volume status was assessed clinically rather than by objective hemodynamic or imaging-based methods. Potential selection bias cannot be excluded. Important variables such as left atrial volume, arteriovenous fistula flow, biomarkers, and invasive hemodynamic measurements were not available.

## Conclusions

In this cross-sectional cohort of 120 patients with stage 3 to stage 5 CKD, echocardiographically defined PH was present in 68 patients, corresponding to a prevalence of 56.7%, with a substantial burden of moderate-to-severe disease. Because PH was classified using estimated PASP derived from tricuspid regurgitation velocity and estimated right atrial pressure, these parameters were not interpreted as independent echocardiographic features of PH. Non-definitional findings associated with PH included advanced CKD, dialysis dependence, volume overload, lower hemoglobin, larger right atrial size, lower tricuspid annular plane systolic excursion, and more frequent diastolic dysfunction. These findings support careful echocardiographic assessment in CKD, but should be interpreted as associative and descriptive rather than causal or externally predictive evidence.
